# Recent Advances in Computer-Aided Structure-Based Drug Design on Ion Channels

**DOI:** 10.3390/ijms24119226

**Published:** 2023-05-25

**Authors:** Palina Pliushcheuskaya, Georg Künze

**Affiliations:** 1 Institute for Drug Discovery, Medical Faculty, University of Leipzig, Brüderstr. 34, D-04103 Leipzig, Germany; palina.pliushcheuskaya@uni-leipzig.de; 2 Interdisciplinary Center for Bioinformatics, University of Leipzig, Härtelstr. 16-18, D-04107 Leipzig, Germany

**Keywords:** ion channels, ion channel structure, drug design, virtual screening, ligand docking, molecular dynamics

## Abstract

Ion channels play important roles in fundamental biological processes, such as electric signaling in cells, muscle contraction, hormone secretion, and regulation of the immune response. Targeting ion channels with drugs represents a treatment option for neurological and cardiovascular diseases, muscular degradation disorders, and pathologies related to disturbed pain sensation. While there are more than 300 different ion channels in the human organism, drugs have been developed only for some of them and currently available drugs lack selectivity. Computational approaches are an indispensable tool for drug discovery and can speed up, especially, the early development stages of lead identification and optimization. The number of molecular structures of ion channels has considerably increased over the last ten years, providing new opportunities for structure-based drug development. This review summarizes important knowledge about ion channel classification, structure, mechanisms, and pathology with the main focus on recent developments in the field of computer-aided, structure-based drug design on ion channels. We highlight studies that link structural data with modeling and chemoinformatic approaches for the identification and characterization of new molecules targeting ion channels. These approaches hold great potential to advance research on ion channel drugs in the future.

## 1. Introduction

**Figure 1:**
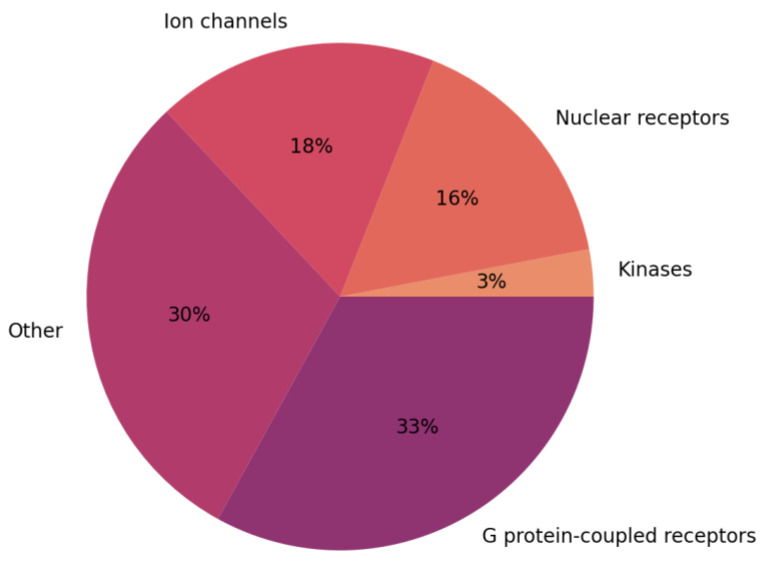
Percentage of drugs targeting major protein families (data from: [7]).

Ion channels are membrane proteins that form pores within cell membranes, allowing the passive transport of ions, such as Na^+^, K^+^, Ca^2+^, and Cl^−^, from one side of the membrane to the other. More than 300 different ion channels, belonging to 13 different families, have been identified in the human genome [1]. Ion channels play important roles in a variety of biological processes, such as electric signaling in excitable cells, the regulation of nutrient transport in epithelial cells, and the maintenance of ion homeostasis in subcellular organelles (e.g., the regulation of calcium levels in the endoplasmic reticulum) [2,3]. Many human diseases are caused by the disruption of normal ion channel function [4,5,6]. Disorders resulting from mutations in ion channel encoding genes, so called channelopathies, include a variety of pathologies of the nervous, cardiovascular, and endocrine system. Because of their central pathophysiological functions, ion channels represent major drug targets and only 18% of currently used drugs target them. This makes ion channels the second largest drug target group (Figure 1) [7].

**Figure 2:**
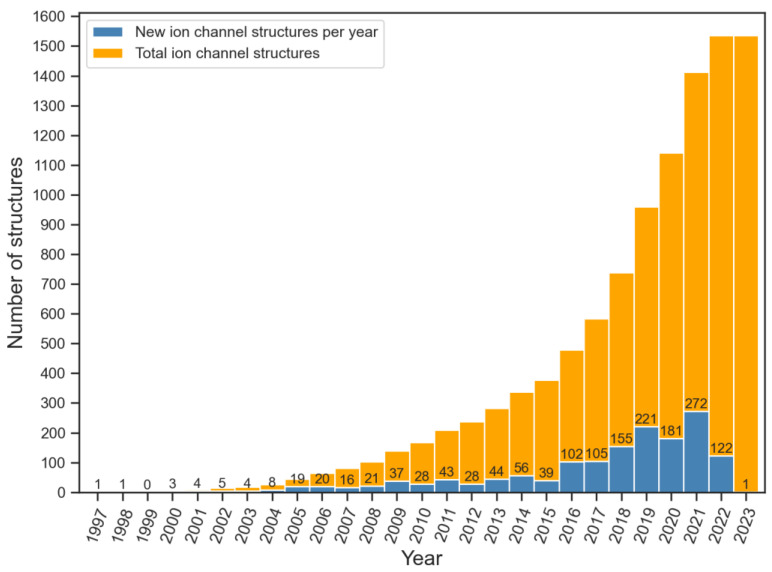
Number of structures of ion channels released every year since 1998 (data were obtained from mpstruct database [9], last look up date: 31 March 2023). Values on top of blue bars indicate the number of new ion channel structures released every year.

In the past 25 years, there has been a significant increase in the number of ion channel structures. Starting from 1998 when the first three-dimensional structure of an ion channel, the KcsA channel from *Streptomyces lividans*, was solved with the help of X-ray crystallography [8], the number of ion channel structures has increased to more than one thousand five hundred [9] (Figure 2). This progress has been primarily driven by technological advancements in X-ray crystallography and cryo-electron microscopy (cryo-EM).

Structure determination of many ion channels in complex, with small molecule ligands, has become feasible as well and ca. 500 unique ion channel-ligand structures have been determined to date [10]. Table 1 lists the number of released structures of ion channels in complex, with small molecule ligands, for major ion channel families classified according to the nomenclature of the Structural Classification of Proteins (SCOP) and Transporter Classification (TCDB) databases [11,12]. Analyses of these structures reveal that drugs target ion channels via diverse binding sites and modulate ion channel function through various modes of action.

The available structures of ion channels at a high resolution have offered valuable understanding of the molecular mechanisms of drugs and their interactions with their target protein. Additionally, these structures serve as an important starting point for the rational structure-based design of ion channel-targeting drugs. As a result, computational techniques for virtual drug screening and structure-based design have now become an increasingly important component of the discovery process. In this review we summarize and highlight recent advances in the development and application of structure-based drug discovery methods and their usage for designing drugs to target ion channel (patho)physiological functions. After outlining the most important ion channel families, their molecular structure, disease mechanisms, and current pharmacological treatment options, we show how different computational drug design approaches have been used to characterize the interactions between ion channels and small molecule ligands to search for new ion channel drugs, and model their structure–activity relationships.

### 1.1. Classification of Ion Channels

Ion channels can be classified by different properties, including their gating mechanism or their ion selectivity. Voltage-gated ion channels can be opened upon changes of the membrane potential, which makes the channel conductive, allowing the passage of ions down their electrochemical gradient. Based on the ion type that is being conducted, human voltage-gated ion channels can be further subdivided into:

- Voltage-gated sodium (Na_V_) channels, which are responsible for the onset and propagation of the action potential in neurons [13].
- Voltage-gated potassium (K_V_) channels, which among other functions, determine the shape and duration of the action potential, control the membrane resting potential, and modulate hormone secretion [14].
- Voltage-gated calcium (Ca_V_) channels, which take part in various processes, including cardiac action potential propagation, muscle contraction, and calcium-dependent gene expression [15].
- Voltage-gated chloride channels (CICs), which contribute to regulating neuronal excitability and cell volume [16].

In addition, the voltage-gated proton channel H_V_1 is conductive for protons, thus regulating the intracellular pH and membrane hyperpolarization [17].

Ligand-gated ion channels are opened by binding of chemical molecules, such as intracellular second messengers (e.g., cyclic guanosine monophosphate (cGMP) and inositol trisphosphate (IP_3_)) or ions (e.g., H^+^). Ligand binding triggers conformational changes in these ion channels, which makes them conductive [18]. The most prominent members of the ligand-gated ion channel family are 5-hydroxytryptamine (5-HT_3_) receptors, nicotinic acetylcholine (nACh) receptors, acid-sensing (proton-gated) ion channels (ASICs), epithelial sodium channel (ENaC), GABA_A_ receptors, glycine receptors, ionotropic glutamate receptors, inositol triphosphate (IP_3_) receptors, purine (P2X) receptors, and the zinc-activated channel (ZAC) [19].

In addition to ion channels that are primarily voltage-gated or ligand-gated, some ion channel families exist that have more diverse modes of activation. Transient receptor potential (TRP) channels, e.g., constitute to a family of cation permeable ion channels that includes 28 different genes in humans, are grouped into six different subfamilies based on amino acid sequence homology (TRPC, TRPM, TRPV, TRPA, TRPML, and TRPP) [20,21]. TRP channels can be activated by different physical and chemical stimuli, including second messenger molecules (PIP_2_, DAG, Ca^2+^), which are produced in signaling pathways downstream of G protein-coupled receptors and receptor tyrosine kinases, as well as changes in membrane voltage, temperature, and osmotic pressure [22]. Two-pore domain potassium (K2P) channels represent another family of potassium permeable channels, comprising 15 members in humans, which are activated by diverse mechanisms including lipids (e.g., arachidonic acid), pH, voltage, small molecules (e.g., anesthetics), and membrane pressure [23].

### 1.2. Mechanism of Action of Ion Channels

**Figure 3:**
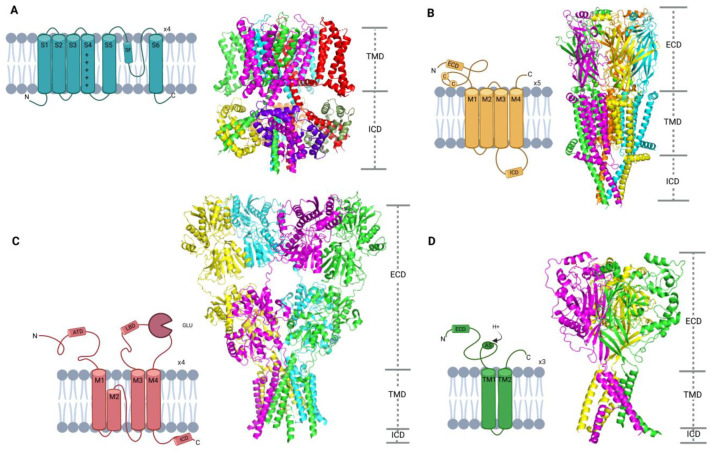
Subunit composition and overall structure of major ion channel superfamilies. A schematic 2D topology diagram is shown on the left in each panel and a representative 3D cryo-EM structure is displayed on the right with the indication of extracellular (ECD), transmembrane (TMD), and intracellular domains (ICD). Each of the subunits is represented in a different color. (**A**) Voltage-gated ion channels consist of 4 subunits or repeats, each with S1–S6 transmembrane helices. The voltage-sensing domain is formed by S1 to S4, and the selectivity filter (SF) by the S5–S6 connecting loop. Positively charged residues in S4 are indicated with a “+” sign (PDB code: 7XNI [37]). (**B**) Cys-loop ion channels consist of 5 subunits, each comprising M1–M4 transmembrane domains, a large extracellular domain (ECD) with a characteristic loop stabilized by a disulfide bond, and a large intracellular domain (ICD) between M3 and M4 (PDB code: 7KOO [38]). (**C**) Glutamate-gated ion channels consist of 4 subunits, each with M1-M4 transmembrane domains, large extracellular amino-terminal (ATD) and ligand-binding (LBD) domains, where glutamate (GLU) binds, and a carboxy-terminal intracellular domain (ICD) (PDB code: 3KG2 [39]). (**D**) Acid-sensing ion channels consist of 3 subunits, each with TM1-TM2 transmembrane domains and a large extracellular domain (ECD), where the activation site (AS) for proton binding is located. N and C represent the N and C termini, respectively (PDB code: 6AVE [40]).

The function of ion channels, their ion selectivity, and their mode of activation and deactivation are tightly linked to their structures. Members of the voltage-gated like (VGL) ion channel superfamily usually have a tetrameric or pseudo-tetrameric topology (Figure 3A) [24]. Each of the four subunits, or repeats, consist of six transmembrane segments (S1–S6). There are three main structural moieties: the voltage-sensing domain (VSD), which is formed by segments S1 to S4 and the connecting linker regions, the central ion-conducting pore domain (PD), which is formed by S5 and S6 of all four subunits, and the ion selectivity filter, which is formed by the S5–S6-connecting pore loop when all four subunits come together. Activation of VGL ion channels (K_V_, Na_V_, TRP, CNG, etc.) results from voltage-driven movement of charged residues in the voltage-sensing domain [25,26]. Several members of VGL ion channels, like cyclic nucleotide-gated ion channels, are also tetrameric and contain all the structural motifs that are present in VGL ion channels. However, the VSD is deprecated in the cyclic nucleotide-gated ion channels; therefore, activation of these channels proceeds upon binding of endogenous ligands (e.g., cGMP or cAMP) [27].

Members of the cys-loop ion channel superfamily (nACh, 5-HT_3_, GABA_A_ and glycine receptors) have a pentameric structure with all subunits pseudo-symmetrically arranged to form an ion conducting pore (Figure 3B) [28]. Each of the five subunits contains a loop of 13 amino acids surrounded by cysteines within the extracellular domain (ECD). The ECD also contains the ligand-binding site, where the activation of cys-loop ion channels takes place. The transmembrane domain includes four alpha-helices (M1–M4) that form the ion-conducting pore. M3 and M4 helices span down to the intracellular domain (ICD) yielding a linker of 50–250 amino acid residues, which takes part in the assembly and trafficking of the cys-loop receptors [29,30].

Glutamate-gated ion channels (AMPA, NMDA, kainate receptors, etc.) are tetrameric ligand-gated ion channels with a large extracellular domain, which is larger than in other ion channels (Figure 3C) [31]. Glutamate receptors consist of the extracellular amino-terminal domain (ATD), the extracellular ligand-binding domain (LBD), the transmembrane domain (M1, M2, M3, M4), and the intracellular carboxyl-terminal domain (ICD) [32]. Activation of glutamate ion channels is characterized by an exclusive binding of the endogenous neurotransmitter glutamate that leads to the opening of the pore region [33].

Acid-sensing ion channels (ASICs) belong to the trimeric proton-gated ion channel superfamily (Figure 3D) [34]. They are characterized by a sizable extracellular domain (ECD), where the acidic activation pocket resides, and two transmembrane domains (TM1 and TM2) for each of the three subunits, together forming the pore domain [35]. Under acidic pH, a proton binds to the activation site in the ECD leading to the conformational changes in the TM2, thus, opening the pore of the channel [36].

## 2. Ion Channels as Drug Targets

The targeting of ion channels with small-molecule drugs or natural toxins is evaluated as treatment option in different diseases, such as neurological and cardiovascular diseases, muscular degradation, and pain sensation disorders [41,42]. Despite that, there are 300 types of ion channels known, and currently available drugs are rarely selective towards a certain ion channel subtype. Additionally, approximately 25% of drugs that target ion channels are being launched after clinical trials, and 75% fail [42]. This highlights the great pharmacological potential of targeting ion channels but also the difficulty in developing new drugs with high selectivity and potency.

This part of the review provides a brief overview of the pharmacologically most studied ion channel families, and of the therapeutics that were developed to target them.

### 2.1. Voltage-Gated Sodium (Na_V_) Channels

There are nine members of the Na_V_ channel family: Na_V_1.1 to Na_V_1.9. They are mostly known for their role in electrical signaling in the cells of the CNS, as well as the neuromuscular and cardiovascular systems [43]. Marketed drugs that are known to act on Na_V_ channels, and block them, include anticonvulsants (carbamazepine, lamotrigine), antidysrhythmics (tocainide, mexiletine), and local anesthetics (lidocaine, benzocaine). All these drug molecules have a neutral or slightly basic pka and bind to a common site in Na_V_ channels, which stems from the fact that all members of the Na_V_ channel family have a high amino acid sequence similarity. This underlines the fact that most of the known Na_V_ channel ligands are not selective towards a certain subtype [43].

### 2.2. Voltage-Gated Potassium (K_V_) Channels

K_V_ channels represent the most diverse and largest ion channel family [44]. Based on sequence homology they are divided into twelve subfamilies, K_V_1–K_V_12, each with one to eight different subtypes. The great amount of K_V_ channel subtypes is expressed in the CNS; thus, these channels are being targeted in neurological diseases, such as epilepsy, episodic ataxia, psychosis, and multiple sclerosis [45,46]. K_V_ channels are also involved in the regulation of cell proliferation and apoptosis, which establishes their role in cancer progression and their targeting in anti-cancer therapies [44].

Among the most known drugs that act on K_V_ channels are the anti-epilepsy drugs retigabine (K_V_7.2) and azimilide, which reduce the duration of cardiac action potential (K_V_7.1) [47], and amifampridine, as a treatment for congenital myasthenic syndromes and Lambert–Eaton myasthenic syndrome (KCNA1) [46].

### 2.3. Voltage-Gated Calcium (Ca_V_) Channels

Just like Na_V_ channels, Ca_V_ channels are present in neurons and excitable cells. They are activated upon membrane depolarization and can be grouped into two major categories based on the voltage change that is required to open them (high- and low voltage-activated) [38]. Additionally, Ca_V_ channels can be divided into five types based on the type of Ca^2+^ current they induce (L-, N-, P/Q-, R-, and T-currents) [48].

Members of the Ca_V_1 subfamily are known targets of antihypertensive and antiarrhythmic drugs, due to their role in regulating the cardiac rhythm. Phenylalkylamines and benzodiazepines also act on Ca_V_1 channels. Ca_V_2 channels are presynaptic calcium channels and mediate the release of neurotransmitters from the presynapse. Drugs that act on Ca_V_2 members (e.g., dihydropyridines) are usually taken to treat epilepsy and migraines. Ca_V_3 subtypes are mostly present in heart muscle cells and serve as targets for vasodilating drugs, such as mibefradil [45,46].

### 2.4. Ligand-Gated Ion Channels

The ligand-gated ion channel superfamily includes ion channels that are activated upon the binding of various neurotransmitters (γ-aminobutyric acid (GABA), glutamate, glycine, 5-hydroxytryptamine (5-HT), etc.) [49]. Because of the variety of neurotransmitters, these ion channels serve as drug targets for various diseases. Anaesthetic agents [50] and drugs for the treatment of Alzheimer’s disease [51] and anxiety [52], act on ligand-gated ion channels.

### 2.5. Transient Receptor Potential (TRP) Channels

TRP channels mediate a plethora of physiological responses, such as pain and temperature sensation, taste, and sensory signaling. Mutations in TRP channels could lead to neurodegeneration, kidney disease, and bipolar disorders [18].

### 2.6. Ion Channel-Drug Interactions

**Figure 4:**
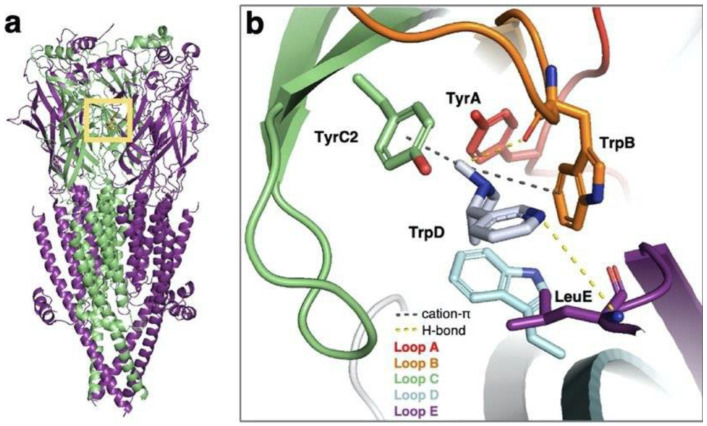
(**a**) Cryo-EM structure of the human nAChR α3β4 receptor (PDB code: 6PV7 [57]) with binding site indicated by a yellow box. (**b**) Close-up view of the binding site with nicotine bound in purple. H-bonds are shown in yellow and cation−π interactions are shown in black. (Reprinted with permission from Ref. [56]. Copyright 2023, American Chemical Society.

Because of the high structural and functional diversity between different ion channel superfamilies, small molecule ligands bind at various locations within their structures. For instance, activation of the voltage-gated potassium channel KCNQ2 proceeds upon binding of different drug molecules, among which are retigabine and ztz240. Although retigabine and ztz240 are both activators of KCNQ2 and exhibit antiepileptic activity, they bind at distinct sites in the channel. Retigabine binds at the pore domain and activates the channel through allosteric modulation, whereas ztz250 was shown to be bound at the VSD, thus directly stabilizing the activated state [53]. Benzodiazepines, which are widely used drugs targeting GABA_A_ receptors and acting as anticonvulsants and muscle relaxants (e.g., diazepam), are known to bind either in the extracellular or in the transmembrane domains [54,55]. Nicotinic acetylcholine receptors, which belong to the cys-loop receptors superfamily along with GABA_A_ receptors, also exhibit an extracellular ligand-binding site. The binding of the endogenous nicotine molecule and agonistic smoking cessation drug cytisine, along with other small molecule agonists, was studied in the nAChR α3β4 subtype to highlight important interactions that can be exploited to enhance drug selectivity (Figure 4) [56].

## 3. Computer-Aided Drug Design Approaches

**Figure 5:**
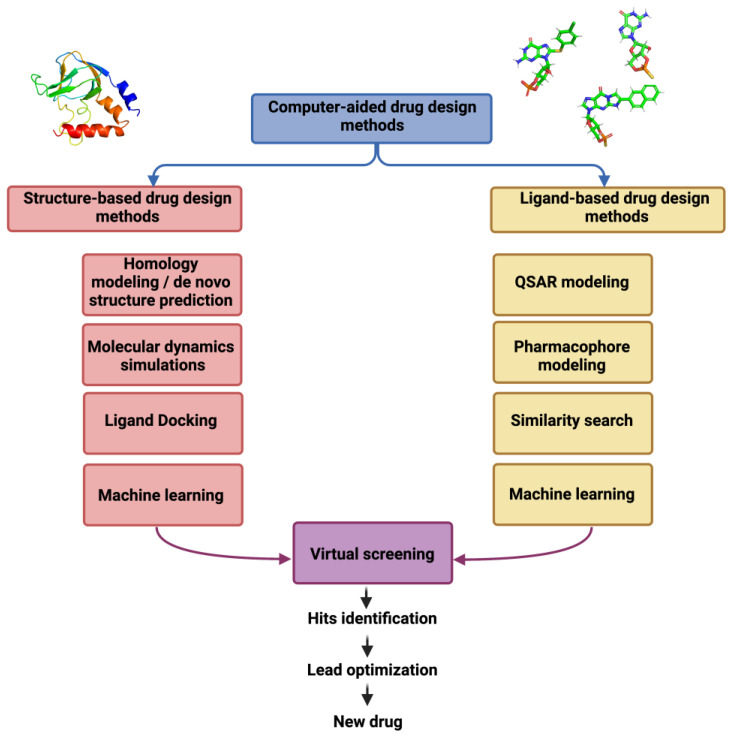
Overview of computer-aided drug design methods and their role in drug discovery.

Computer-aided drug design (CADD) has become an essential part of modern drug discovery pipelines. CADD methods can help in identifying new lead compounds, predicting drug activity and biological properties, and modifying an existing drug to improve its therapeutic action or avoid side-effects [58]. Thus, CADD methods help to speed up the whole drug development pipeline and entry into clinical testing of a newly developed compound [59]. Generally, CADD is classified into structure-based drug design and ligand-based drug design methods (Figure 5), which can be combined with each other and with experimental techniques to promote an iterative drug optimization [60].

### 3.1. Structure-Based Drug Design

Structure-based drug design (SBDD) methods make use of the protein three-dimensional structure [61], which can be obtained with the help of X-ray crystallography [62], NMR spectroscopy [63], or cryo-EM [64]. In the absence of a protein three-dimensional structure, homology modeling or de novo structure prediction techniques can be used to determine the three-dimensional structure computationally [65]. In particular, deep learning-based methods, such as AlphaFold [66], have led to a major breakthrough in protein structure prediction, expanding the range of proteins for which a reliable protein structure model can be constructed. Still, whether the accuracy of the computational model in the region of the ligand binding pocket is sufficient for structure-based drug design efforts, has to be evaluated on a case-by-case basis. Afterwards, potential drug molecules can be virtually docked into the ligand-binding pocket to identify molecules that could bind to the target protein and predict their modes of interaction [59,67].

The accuracy of docking models depends on scoring functions, which are implemented in docking softwares to analyze protein–ligand interactions. Scoring functions can be classified as physics-based, knowledge-based, empirical, and machine learning-based [68,69]. Each of them provides a certain representation of the free energy of binding of a ligand. Physics-based scoring functions rely on force field parameters that are retrieved from experimental data and quantum mechanical calculations [70]. Knowledge-based scoring functions approximate the binding energy with the help of distance-dependent pairwise potentials that are derived from statistics on experimentally determined complex structures by applying the Boltzmann law [71]. Empirical scoring functions evaluate the binding affinity of a ligand by summing up the energetic contributions of a protein–ligand complex, including hydrogen bond energy, Van der Waals energy, solvation energy, and others [72]. Finally, with the significant improvement of computational power, there are also machine learning-based scoring functions that are developed by training a machine learning model on a dataset of known binders and non-binders to predict the binding affinity for a new ligand. Although, they usually outperform other types of scoring functions, machine learning scoring approaches can be challenging to integrate into existing docking softwares and can suffer from problems such as over-fitting and poor generalizability [73]. Depending on a docking program, different types of scoring functions are used to rank docking decoys by their docking scores. Top rated models are usually compared to the experimentally determined complex structure, if available, or to other good scoring models in terms of their RMSD values, to evaluate the amount of sampling and convergence of the docking calculation [74]. Furthermore, the research community proposes suitable scores to be used for a particular docking software to estimate the likelihood that a docking pose represents a true ligand binding event [75].

Docking protocols can be supplemented by the use of pharmacophore modeling. A pharmacophore model describes interactions that a potential ligand is expected to have and that are relevant for binding and activity. When docking is combined with pharmacophore models, it may identify more reliable active molecules by ignoring ligands with undesirable interaction patterns [76,77]. Docking programs have been developed that can easily incorporate pharmacophore models in docking (e.g., PharmDock) [78].

Furthermore, docking can be assisted by the help of deep learning to speed up the docking procedure and allow to virtually screen billions of molecules in limited time. Particularly, the deep docking method utilizes deep neural network (DNN) processing of large libraries of chemical compounds. Only a small fraction of molecules are being conventionally docked, while docking scores of the remaining compounds are being predicted by the DNN, which was pre-trained on the above-mentioned small ligand subset. An advantage of this DNN-based docking protocol is that it can be integrated in different docking softwares [79,80].

Molecular dynamics (MD) simulations also belong to the SBDD methods and are employed to study the structural dynamics of the protein–ligand complex at atomic detail, and obtain the thermodynamic and kinetic parameter for the protein–ligand interaction. Possible use cases of MD in structure-based drug design includes the generation of protein conformational ensembles for docking, which better represents the flexibility of the ligand binding pocket, the study of the binding pathway, on which a ligand approaches its target protein, and in combination with other analyses, the assessment of relative free binding energies and ligand dissociation rates [81]. With umbrella sampling, ligand-binding affinities may be estimated. The procedure employs a calculation of potentials along the obtained reaction coordinates, which are meant to cancel energetic barriers, thus facilitating extensive sampling of conformational changes [82,83,84]. Ligand dissociation rates or ligand residence times can be determined with, e.g., random acceleration molecular dynamics (RAMD) [85], its improved version τ-random acceleration molecular dynamics (τRAMD) [86], and other methods. In τRAMD an additional force is applied to the ligand to trigger the unbinding process, from which the relative k_off_ rate of ligands can be calculated. It allows ranking ligands by their relative k_off_ rates and requires a reduced time spent on the simulation itself [85]. In case of metadynamics simulations, which are widely applied to the study of ion channel transition states and free energy calculations, machine learning techniques can help to derive collective variables that describe the essential dynamics of a molecular system of interest, thus shortening the simulation time in metadynamics. Principal component analysis, variational autoencoders, and various classifiers (logistic regression, support vector machines, etc.) are used as dimensionality reduction methods to infer collective variables [87,88].

### 3.2. Ligand-Based Drug Design

The ligand-based drug design (LBDD) approach does not rely on a protein three-dimensional structure but analyzes the structures and chemical properties of known active and inactive small molecules, with the aim of relating these features to the biological activities of the molecules. With the help of chemoinformatics tools, molecular descriptors for a set of molecules can be derived that are correlated with their therapeutic effects using mathematical modeling [89]. The developed quantitative structure–activity relationship (QSAR) model can then be used to predict the activity of a potential therapeutic compound and virtually screen small molecule libraries with the goal to identify new compounds with similar or improved activity [90]. One can also specify a pharmacophore model as an input to the virtual screening pipeline to guide the search towards molecules with desired chemical and structural properties [91]. A pharmacophore represents a set of steric and electronic features of a molecule, such as conserved functional groups common to all hit compounds, or hydrogen bond donor/acceptor atoms that are necessary to ensure an optimal interaction with the target macromolecule.

Machine learning (ML) can be applied in QSAR modeling to predict the biological properties of molecules. By learning the patterns in the training dataset, where compounds are defined by a set of descriptors, various machine learning methods can classify and predict the ligand of interest in context of its activity, toxicity, and other biologically relevant features [90,92]. Support vector machines (SVMs), random forest (RF) and k-nearest neighbors (KNNs) algorithms are widely used for classification purposes. SVMs, in particular, are useful to deal with high-dimensional data, where they map an unknown compound to a specific category. RF has also found its place in the drug design field. It is based on decision trees, where it combines each decision to make a prediction. The final label is averaged over all predictions, thus assigning a molecule to a particular class [92,93]. The KNN classifier is similar to RF, with classifications made by utilizing a majority rule. Thus, a molecule is assigned to a category based on the category of the closest neighbors within a certain distance [90].

Several ML-based QSAR models dedicated to ion channels have been made available as webservers. For instance, Pred-hERG is a computational tool which predicts whether a compound is a blocker of the human-ether-a-go-go-related gene (hERG) channel. The algorithm utilizes SVMs and provides the user a probability of hERG blockage based on the input molecule in SMILES format [94,95,96]. The vNN-ADMET webserver predicts the important absorption, distribution, metabolism, excretion, and toxicity (ADMET) properties of molecules. By specifying a query in a SMILES format, the nearest neighbors algorithm is applied to predict whether a compound complies with the ADMET rules, which a successful drug candidate should do in order to pass clinical trials [97,98]. The availability of these webservers is particularly useful at the early stages of drug design campaigns. Their output can provide a set of guide molecules that pass, at least, the necessary requirement.

LBDD strategies are also being augmented with deep learning techniques. Deep recurrent neural network (RNN), represented by a deep long short-term memory network, was applied to obtain hit drug molecules with the desired profile according to the project requirements [99]. Conditional RNN utilizing information on a ligand-binding pose, generated molecules that exhibited a desired binding mode in contrast to traditional medicinal chemistry approaches [100]. Although deep learning techniques usually outperform conventional methods, it is important to use high-quality training data and tune the network properly to achieve a necessary performance.

### 3.3. Virtual Ligand Libraries for Ion Channel Drug Discovery

There is a variety of small molecule datasets available that can be used in screening for new active compounds, as well as for the exploration of QSAR and ADMET properties. One of the most comprehensive and commonly used datasets is the ChEMBL database [101]. It is an open-source, web-based database, which contains a substantial amount of data on protein targets and small molecule ligands with associated activity data. The ChEMBL database includes currently data for ~2.3 million ligands and ~15,000 targets, which were collected from published assays, scientific publications, or gathered from other databases and research institutions across the world [102,103]. Screening experiments on ion channels often exploit the ChEMBL ion channel dataset [104,105]. One can specify a particular ion channel family and subtype and obtain a list of small molecule ligands that are known to act on these targets. Several research groups developed workflows and instruments to ease the exploration of the ChEMBL database [106,107,108], some of them are specific for ion channels [109].

Another resource is the DrugBank database, which is also available via a web server and contains around 500,000 drug entries with the respective protein targets and activity information [110,111,112]. Scientific groups utilize the DrugBank database to develop new tools for computer-aided drug design and to search for active molecules for their particular targets of interest. In particular, Feng et al. [113] developed a machine-learning model to screen the DrugBank database to identify blockers of the hERG channel and assess their likelihood of causing cardiotoxic effects. Wu et al. [114] described several neural network based methods to predict drug–target interactions, which was evaluated on the DrugBank database and supplied hit molecules for subsequent biological assessment.

In addition to the ligands of ion channels in ChEMBL and DrugBank datasets, subsets of ion channel-targeting molecules exist also within the web-based KEGG database [115] and the DUDE online decoy generator [116]. There is also a large number of commercial ion channel libraries provided within such suites, e.g., Enamine [117], ZINC database [118], ChemBridge [119], and Life Chemicals [120].

## 4. Computational Drug Design on Ion Channels: Recent Discoveries

### 4.1. Computational Refinement of Cryo-EM Structures

As mentioned above, structure-based CADD methods require a three-dimensional structure of the protein target of interest. However, not for every protein has a three-dimensional structure been determined. Several tools exist for protein structure prediction. Homology modeling employs information from template structures of homologous proteins. Some common tools include SWISS-MODEL [121], RosettaCM [122], and Modeller [123]. However, with the advance in the artificial intelligence field, these methods were superseded by methods like AlphaFold [66], RoseTTAFold [124], or OmegaFold [125], which can accurately predict protein three-dimensional structures in the absence of template information and also provide a likelihood of the structure accuracy.

AlphaFold can also be used to refine protein models obtained from cryo-EM density maps. Terashi et al. [126] developed a deep learning-based model quality score that evaluates the likelihood of an amino acid residue’s position in the cryo-EM map. The regions of the protein that are classified as incorrect, according to this score, are subjected to remodeling with AlphaFold to generate a refined structure. The protocol was tested on membrane proteins and can be used to refine structures of ion channels.

**Figure 6:**
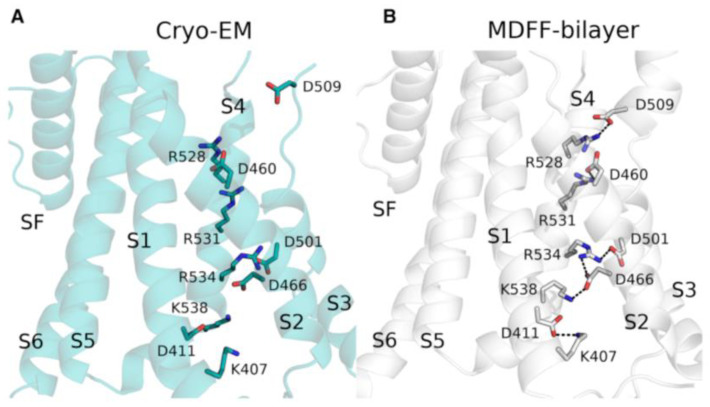
Functionally important salt bridges in the hERG structure by Khan et al. [127]. (**A**) Starting cryo-EM structure. (**B**) Structure after fitting refinement. (Reprinted (adapted) from Ref. [127]. Copyright 2021, Elsevier.

Furthermore, Khan et al. [127] used MD simulations to refine the cryo-EM structure of the hERG ion channel. The hERG gene encodes a voltage-gated potassium channel (K_v_11.1), wherein mutations are the main cause of long QT syndrome [128]. The cryo-EM structure of the human hERG channel was solved at medium resolution (3.8 Å) [129] and the data from the structure, in particular the positions of amino acid sidechains, were inconsistent to numerous experimental studies highlighting the importance of salt bridges and other interactions present in the VSD. Therefore, the structure was refined using the molecular dynamics flexible fitting (MDFF) method [130]. The bilayer-embedded structure, used in fitting, was obtained from a long MD simulation of the hERG channel structure of several microseconds. The refined structure showed the presence of salt bridges in the VSD that were known from experimental studies to be important for channel function (Figure 6). Additional salt bridge interactions were detected in the structure and confirmed in electrophysiological testing (Figure 6).

MD simulations were also used to refine the cryo-EM structure of the glycine receptor [131]. Glycine receptors belong to the family of pentameric ligand-gated ion channels and are targets for chronic pain treatments [132]. Dämgen et al. [131] performed all-atom MD simulations to refine the open state cryo-EM structure of the glycine receptor and described several interactions that stabilize the open state.

### 4.2. Characterization of Drug-Ion Channel Interactions by Molecular Dynamics Simulations

**Figure 7:**
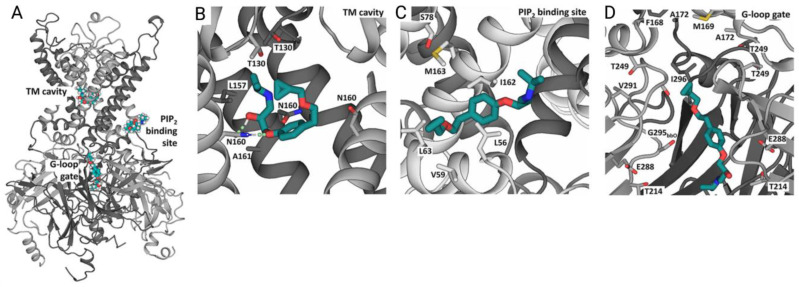
Betaxolol binding sites in K_ATP_ channel identified by Houtman et al. [133]. (**A**) Overview of betaxolol binding sites. (**B**–**D**) Zoom-in views of betaxolol binding sites at (**B**) TM cavity, (**C**) PIP_2_ binding site, (**D**) G-loop gate. (Reprinted (adapted) from Ref. [133]. Copyright 2022, Frontiers.

MD simulation is a widely used method in drug design, which can be used to investigate, e.g., how a potential active molecule influences ion channel gating and ion conduction processes. Houtman et al. [133] and Chen et al. [134] used MD coupled with pharmacophore modeling to identify new inhibitors of ATP-dependent potassium channels Kir6.1 and Kir6.2. Obtaining function mutations in K_ATP_ channels lead to the development of epilepsy and neonatal diabetes, known as the DEND syndrome. Existing drugs derived from sulfonylurea inhibit K_ATP_ channels but fail to elicit significant inhibition of disease-causing K_ATP_ channel mutants, which are known to be sulfonylurea resistant [135]. Thus, with the help of Martini 2.2-based [136], coarse-grained MD simulations, putative binding sites of the hit molecule betaxolol were identified. Three main binding sites were discovered and tested in more detail in all-atom MD simulation in Gromacs (Figure 7). In subsequent all-atom simulations, betaxolol was found to interact with a set of pore-lining residues in the protein, thus plugging the ion conduction pathway and explaining its mode of action. Complexes with betaxolol were tested in inhibition studies by the patch–clamp technique and showed inhibition (IC_50_) values of 27–37 μM towards the K_ATP_ channel. Moreover, betaxolol was also shown to inhibit sulfonylurea-resistant DEND-causing K_ATP_ channel mutants.

Yelshanskaya et al. [137] applied MD-based experiments to study the binding of trans-4-butylcyclohexane carboxylic acid (4-BCCA), which acts as an inhibitor of α-amino-3-hydroxy-5-methyl-4-isoxazolepropionic acid (AMPA) receptors. Overactivation of the AMPA receptors results in seizure occurrences and epilepsy. Perampanel is known to inhibit AMPA receptors but may cause behavioral side effects [138,139]. In this study, interactions between the 4-BCCA and AMPA receptor at the lateral portals formed by transmembrane segments M1–M4 were identified. The all-atom MD simulations indicated that the binding of 4-BCCA is very dynamic with multiple ligand poses entering the channel pore, and that binding may occur simultaneously with perampanel. Thus, it was postulated that 4-BCCA may act as a synergetic inhibitor taken together with perampanel.

Shi et al. [140] employed all-atom MD simulations to analyze the mode of action of zafirlukast on the Ca^2+^-activated chloride channel TMEM16A. TMEM16A is a drug target against lung adenocarcinoma [141]. Zafirlukast was identified as a potential drug candidate through a virtual screen of 1400 FDA-approved drugs. Using MD simulations, the authors showed that zafirlukast blocks the pore by binding to the nonselective inhibitor binding pocket of the TMEM16A channel. Zafirlukast inhibited the proliferation of lung adenocarcinoma cells in vitro and could significantly decrease tumor growth in mice in vivo. It was also assigned a better safety and stability profile compared to natural compounds against lung adenocarcinoma discovered by the same group.

Zimova et al. [142] used molecular docking and MD tools to characterize the binding site of the drug duloxetine on the TRPC5 ion channel. Duloxetine is used to treat severe painful disorders that are difficult to manage in clinical practice [143]. TRPC5 is a cold-sensitive, calcium-permeable, non-selective cation channel, which is known to be involved in the development of painful neuropathies [144]. The authors established that duloxetine suppresses TRPC5 action, which explains its analgesic effect. The molecular docking and all-atom MD simulation results suggested that duloxetine binds to the VSD in TRPC5, which was subsequently confirmed by point mutagenesis.

Drug repurposing is a strategy that seeks to find known drugs for the treatment of diseases that are outside of the original medical indication [145]. This strategy has also been applied in the development of drugs against COVID-19. Toft-Bertelsen et al. [146] demonstrated that amantadine and hexamethylene–amiloride are able to inhibit ion channel activity of Protein E from SARS-CoV-2. Amantadine is a well-known ion channel blocker and has been used already for several decades for the influenza treatment [147]. Toft-Bertelsen et al. used all-atom MD simulations to characterize the binding profile of amantadine, and observed that it binds to the amino acid residues in the pore region, thus blocking the current in Protein E and inhibiting its activity that directly relates to the virus replication and subsequent inflammation.

### 4.3. Identification of Ion Channel Ligands by Virtual Docking Techniques

Drug design endeavors require detailed understanding of how a small molecule binds to the target of interest. To achieve high binding affinity and selectivity, a ligand should ideally have a shape, charge, and hydrogen bond donor/acceptor pattern that complement those features in the receptor binding pocket. Molecular docking techniques try to predict low-energy, ligand-binding poses and are massively employed for in silico drug screening [148].

Abdelsayed et al. [149] conducted a virtual docking study combined with validation by electrophysiology to detect molecules that will bind to and restore the function of variants of the voltage-gated sodium Na_V_1.5 channel carrying loss-of-function (LoF) mutations. Na_V_1.5 is a cardiac ion channel and mutations in the Na_V_1.5 gene are associated with various arrythmias [150]. The majority of the existing Na_V_1.5 drugs (e.g., lidocaine [151], ranolazine [152]) target gain-of-function mutants of Na_V_1.5 by blocking the ion channel pore [153]. However, no ligands that will activate and rescue LoF mutants of Na_V_1.5 exist [154]. Abdelsayed et al. [149] performed virtual docking using the ZINC database and obtained 21 hits including molecules with sulfonamide and carboxamide groups. These small molecules were found to bind in side fenestrations of the Na_V_1.5 channel structure, leaving the central ion conduction pathway passable, which is essential for their activity enhancing action. Based on favorable affinity for the fenestrations, six compounds with a hydrophobic core were selected from the initial 21 hits and screened against the Na_V_1.5 channel. The experiments suggested that these six molecules have a higher affinity to fenestrations, thus representing a potential for treating LoF-arrythmias.

Nicotinic acetylcholine receptors (nAChRs) are ligand-gated ion channels that are involved in a variety of biological processes and neuronal disorders, e.g., schizophrenia, epilepsy, and Alzheimer’s disease (AD) [155]. For example, it was shown that the α4β2- and α7-subtypes of nAChRs possesses a high affinity towards the Aβ peptide, which is known to be a major causing factor for the onset and progression of AD. Thus, interactions between the α4β2 and α7 nAChRs and Aβ oligomers may induce malfunction of the synaptic neuron transfer [156,157]. Batista et al. [158] designed a docking-based pharmacophore model that could be used to search for new nAChR inhibitors. The authors started by performing docking of known nAChR ligands (~200 molecules) with available bioactivity data and extensively described critical contacts and binding modes to obtain a pharmacophore model. The sum of contacts present in the docking models were correlated with the biological activity of known ligands, and the model was used to predict the pKi values for each new molecule. The pharmacophore models were validated using ZINC and ChEMBL databases. Approximately 1500 decoys were determined during the validation procedure, which showed compliance with the pharmacophoric maps. Receiver operating characteristic (ROC) values lied suitably within 0.86–0.93 range.

Doñate-Macian et al. [159] performed a large-scale docking experiment on the transient receptor potential vanilloid 4 (TRPV4) cation channel. TRPV4 takes part in numerous biological processes in the human organism. It is involved in the decrease of the regulatory volume in the respiratory epithelium, in the generation of the myofibroblast, and in the development of fibrosis. Thus, mutations or irregularities of the TRPV4 function may lead to asthma development and lung fibrosis. Furthermore, normal TRPV4 functioning is important to fight toxic substances and pathogens [160]. Doñate-Macian et al. [159] performed a docking-based virtual screening of the NCI database [161], consisting of ca. 250,000 compounds, on the TRPV4 channel structure. Based on the docking scores, 40 hit molecules were identified, which were subjected to the evaluation of their biological effect on TRPV4. Three out of forty hit compounds showed an inhibition activity against TRPV4. These three compounds could be further exploited for the development of therapeutics for an antiviral strategy via TRPV4 [162].

### 4.4. Prediction of Ion Channel Ligands with Structure-Based Virtual Screening

Electrophysiological experiments, (e.g., patch-clamp measurements) or fluorescence-based assays, are often used for ion channel screening purposes [163]. However, screening a huge number of molecules by in vitro experiments is not only slow, but also highly expensive. This is why virtual screening represents an approach to compensate for the drawbacks of electrophysiological testing [164].

Liu et al. [165] carried out a docking-based virtual screening experiment to identify blockers of Shaker voltage-gated potassium (K^+^) channels. Blocking of the K^+^ channels’ pore is a widespread approach to design drugs with antiarrhythmic action [47]. New K^+^ blockers were identified by performing a structure-based virtual screening of the MDL Available Chemicals Directory (ACD) database [166] consisting of ~600,000 commercially available chemicals. First, Liu et al. selected 300 compounds out of the ACD database which exhibited the best docking scores. Afterwards, 20 out of 300 candidates were selected according to the drug-like requirements (logP, structure optimization, etc.). These 20 molecules were then tested in electrophysiological assays with six compounds showing an inhibition of voltage-activated K^+^ current. Furthermore, Liu et al. calculated binding free energies of the final six compounds, which were also in agreement with their inhibitory potencies (IC_50_ values).

Structure-based virtual screening was also applied in the study of Pegoraro et al. [167] to identify blockers of the K_V_1.3 ion channel, which is known to be a target in autoimmune diseases and T-cell proliferation [168]. Pegoraro et al. screened a compiled database of 3.3 million commercially and virtually available compounds, and 500 molecules were selected after the docking and optimization procedures. Blocking activity was found for 37 compounds using patch-clamp technology. The study identified molecules exhibiting a new structural biaryl core that exert an inhibitory effect on T-cell proliferation.

**Figure 8:**
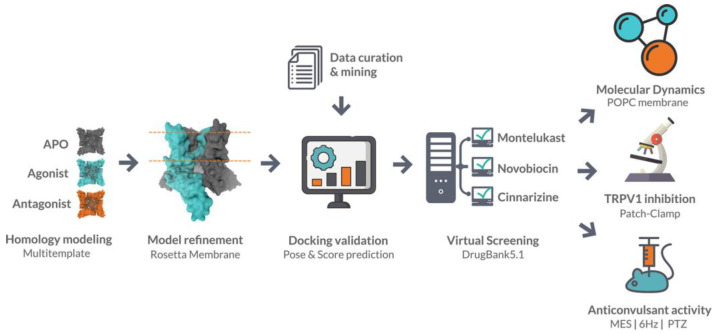
Drug discovery workflow performed by Llanos et al. [169] within a drug repurposing approach to identify inhibitors of the TRPV1 channel. (Reprinted with permission from Ref. [169]. Copyright 2022, American Chemical Society.

Llanos et al. [169] performed a virtual screening on the transient receptor potential vanilloid 1 (TRPV1) receptor as a part of drug repurposing campaign. TRPV1 is a nonselective cation channel that is known to regulate the body temperature [170]. Several TRPV1 antagonists were found to have anticonvulsant activity [171], but because of their hypothermic effect, the development of these types of drugs was hindered [172]. Llanos et al. [169] designed a docking model and applied it to screen the DrugBank database containing over 10,000 approved drugs (Figure 8). They obtained three hit scaffolds represented by montelukast, novobiocin, and cinnarizine molecules. All three molecules showed nanomolar inhibition against TRPV1 and antiseizure activity in in vivo assays.

Using structure-based virtual screening, Pasqualetto et al. [173] identified novel antagonists of the P2X purinoceptor 7 (P2X_7_). P2X_7_ belongs to the family of ligand-gated ion channels, which are activated upon the binding of ATP. P2X_7_ receptor stimulates inflammatory and infection processes in the human body [174]. By performing docking-based screening of the Specs library [175] on the P2X_7_ structure, 17 hit molecules were identified and subjected to evaluation of their biological activity. Among them, compound GP-25 demonstrated an inhibition activity in the micromolar range. Pasqualetto et al. deduced a pharmacophore model based on the interaction pattern between the GP-25 ligand and the P2X7 receptor. The model was then used to screen commercially available GP-25 analogues yielding several compounds with P2X_7_ antagonistic activity.

Virtual screening can be further facilitated by the use of machine learning methods, which have become very popular these days due to advancements in the mathematical theory on machine learning and computational resources [176]. Mostly ligand-based virtual screening approaches are aided with machine learning. For example, Kong et al. [105] designed multiple machine learning frameworks based on ligand molecular fingerprints to screen the ChEMBL database to identify inhibitors of Na_V_1.5 as potential anti-arrhythmic drugs.

## 5. Conclusions and Further Perspectives

Computational methods provide a significant contribution to the drug design field in general, and to ion channel drug discovery in particular. More and more examples of new drug molecules designed with the help of computational approaches have been reported. In silico methods speed up the whole development process and decrease associated costs. Considering the fact that ion channel structures are complicated, having sizable domains that span the membrane and protrude into the extra- and intracellular spaces, further progress in simulation techniques will be needed to help elucidating the channel conformational changes that are occurring over time. This provides critical insight into how a small molecule can modulate the activation process. Future computational advances may provide the opportunity to simulate ion channels for a longer period of time that will, subsequently, provide a more detailed picture of modulation possibilities.

Moreover, currently available drug molecules that act on ion channels, do not possess the desired selectivity. Although the structure itself is complicated, most of the ion channel types have a similar set of elements within it, such as voltage sensor domain, transmembrane helices, gating filter, etc. This is why the development of an active and selective molecule is quite challenging. Machine learning methods are widely used to overcome the selectivity issue by establishing more robust quantitative structure–activity relationships of ion channel ligands. Recent breakthroughs in this field will improve predictions and support design strategies.

Artificial intelligence-assisted, automated protocols are emerging to aid the ion channel drug design field. For instance, the described deep docking protocol can predict the docking scores of millions of molecules [80]. All of these new discoveries will advance ion channel drug research and can lead to effective treatment options in the future.

## Figures and Tables

**Table 1:** Number of total and ligand-bound available structures of ion channels with at least 4 structures present (data from mpstruct, SCOP and TCDB databases [9,11,12]).

Ion Channel Family	Number of Structures	Number of Structures in Complex with Ligands
Transient receptor potential channels	222	165
KcsA voltage-gated K^+^ channels	61	51
Cyclic nucleotide-gated ion channels	53	45
Inward rectifier potassium channels	53	51
Two pore Na^+^ channels	46	45
NaK channels	36	22
Ryanodine receptors	26	25
K^+^ channels MthK	24	16
Two pore Ca^2+^ channels	23	18
Voltage-sensitive calcium channel	22	22
Polycystin cation channels	21	12
KCNQ voltage-gated potassium channels	19	17
ATP-sensitive K^+^ channels	16	15
Slo potassium channels	14	9
Voltage-sensing proton channel	6	3
Calcium-activated SK potassium channels	4	4
Plant inwardly rectifying potassium channels	4	4

## Data Availability

Data sharing not applicable.
